# The contact process on scale-free networks evolving by vertex updating

**DOI:** 10.1098/rsos.170081

**Published:** 2017-05-24

**Authors:** Emmanuel Jacob, Peter Mörters

**Affiliations:** 1 Unité de Mathématiques Pures et Appliquées, Ecole Normale Supérieure de Lyon, Lyon, France; 2 Mathematical Sciences, University of Bath, Bath, UK

**Keywords:** SIS epidemic, spread of infections, temporal network, power-law network, random graph, metastability

## Abstract

We study the contact process on a class of evolving scale-free networks, where each node updates its connections at independent random times. We give a rigorous mathematical proof that there is a transition between a phase where for all infection rates the infection survives for a long time, at least exponential in the network size, and a phase where for sufficiently small infection rates extinction occurs quickly, at most polynomially in the network size. The phase transition occurs when the power-law exponent crosses the value four. This behaviour is in contrast with that of the contact process on the corresponding static model, where there is no phase transition, as well as that of a classical mean-field approximation, which has a phase transition at power-law exponent three. The new observation behind our result is that temporal variability of networks can simultaneously increase the rate at which the infection spreads in the network, and decrease the time at which the infection spends in metastable states.

## 1. Introduction

The spread of disease, information or opinion on networks has been the concern of extensive research over the past decade. Beyond a large body of empirical research and simulation studies, there is now also a growing number of analytic results, based on relatively simple mathematical models of the network and the spreading mechanism [1–4]. A popular assumption on the networks in which a disease is transmitted is that they are scale-free, which means that as the network size grows, the proportion of nodes of degree *k* stabilizes to a limit which, as *k* increases, decays like *k*^−*τ*^ for some positive power-law exponent *τ*. Power-law exponents have been statistically estimated for a range of networks, and there is an abundance of tractable mathematical models for scale-free networks [5–9]. An established model for the spread of an infection on a network is the contact process, or SIS model. In this model, every node can either be infected or healthy. An infected node passes the infection at a fixed rate λ to each of its neighbours, and recovers with a fixed rate of one. Once it has recovered, it is again susceptible to infection.

The extinction time of this type of infection on a scale-free network has been the subject of a controversial discussion in the literature. Pastor-Sattoras & Vespignani [10–12] have used a non-rigorous mean-field calculation to predict a phase transition in the behaviour of the contact process. They predicted that, for networks with a power-law exponent *τ*<3, the infection can survive for a time exponential in the network size regardless of the infection rate. If *τ*>3, however, for sufficiently small infection rate, the time to extinction is at most polynomial in the network size. Chatterjee & Durrett [13] have shown that this prediction is inaccurate and the infection survives for an exponential time for *all* infection rates and power-law exponents. The independent earlier works of Berger *et al.* [14], and of Ganesh *et al.* [15] also give partial versions of this result. Refinements can be found in Mountford *et al.* [16,17]. The failure of the approach of Pastor-Sattoras & Vespignani [10–12] highlights the need for mathematically rigorous arguments in this area of complex networks.

Up to now, rigorous mathematical research has focused almost exclusively on networks that remain constant while the states of the vertices change. In reality, however, connections between individuals change over time, and investigating the effect of this temporal variability on processes taking place on the network is a question of fundamental importance. The study of evolving, or temporal, networks has been identified as an important direction for research, for example, in the concluding paragraph of Durrett [2] and in the recent survey [18]. This paper is intended as a first step into territory almost untouched by rigorous mathematics up to now, offering a rigorous analysis of the contact process on a very simple model of an evolving scale-free network.

When setting up our model, we make three assumptions, which may be justified, for example, if the network is a human interaction network, the updating models movement or migration of individuals, and infection does not greatly affect mobility of individuals. *First*, we assume that the time evolution of the network happens independently of the infection process. This is mainly a simplifying assumption in the light of substantial additional difficulties coming from mutual dependence of processes in random environments.^1^ *Second*, we assume that the evolution of the network and the evolution of infection are on the same time scale, which leads to the most interesting interaction between the evolutions. And *third,* we assume that the power of a node, which varies greatly between nodes in scale-free networks, is not changed over time.

In the absence of individual features of the nodes of the network, there are two natural graph evolutions to study: *edge updating* in which the presence or absence of every edge is updated with a fixed rate, and *vertex updating* in which every vertex refreshes all its connections with a fixed rate. Edge updating has been studied under the name of *dynamical percolation* on a range of regular graph models [24,25]. In our scenario, edge updating would not produce qualitatively different behaviour from the static case, which is why we focus our attention on the vertex updating rule. In line with our assumptions, vertex updating can serve as a rough model for migration of nodes to a different location.

Our main interest in this paper is in the emergence of qualitatively new phenomena owing to the temporal variability of the network. Observe that it is not *a priori* clear whether temporal variability increases or decreases the extinction time of the infection. Our analysis shows that, on the one hand, the temporal variability is helping the system to exit metastable states, thus *decreasing* the extinction time. As a result, beyond a critical value of *τ*, a phase of fast extinction emerges which is not present for the static model. On the other hand, time variability increases the number of nodes infected by a single node, leading to a faster spread of the infection, thus *increasing* the extinction time. As a result, the transition between the phases of fast and slow extinction does not occur at the value *τ*=3, as in the mean-field calculation [11], but at the larger value *τ*=4. This constitutes an interesting new effect emerging in evolving networks.

## 2. Statement of the result

We consider an evolving network model (*G*_*t*_)_*t*≥0_, a family of graphs indexed by continuous time. Its set of vertices is fixed, for all *t*≥0, and identified with a set of labels *V* ={1,…,*N*}. The labels correspond to the strength of vertices, with low labels corresponding to powerful vertices. At time *t*=0, each unordered pair of vertices {*x*,*y*}⊂*V* independently forms an edge with probability

$$p_{x,y}=\min\left\{\frac{\beta N^{2\gamma -1}}{x^{\gamma }y^{\gamma }},1\right\},$$

where *β*>0 and *γ*∈(0,1) are the parameters of the model. This is a special case of the Chung-Lu model, and it is shown in [6] that *G*_0_ is a scale-free network with power-law exponent *τ*=1+1/*γ*. Moreover, the connection probabilities are asymptotically the same as in all rank-one models including the configuration model [6,8,9], and we expect the same qualitative behaviour for all these models. Note that the expected degree of the vertex labelled *x* is proportional to (*N*/*x*)^*γ*^, reflecting the strong hierarchy of vertices. We remark that, unless $\gamma =\frac{1}{2}$, the connection probability differs from that in the preferential attachment models [7] and a further analysis not included here shows that the results of this paper do not extend to that class.

The time evolution of the edges of the network obeys the following rule. Each vertex updates independently with intensity *κ*>0. When vertex *x* updates, first all adjacent edges are erased and then every unordered pair {*x*,*y*}⊂*V* , for *y*∈*V* ∖{*x*}, forms an edge with probability *p*_*x*,*y*_, independently of its previous state and of all other edges. The remaining edges {*w*,*y*} with *w*,*y*≠*x* remain unchanged. Note that the network evolution is stationary, namely for every *t*≥0, the law of *G*_*t*_ is equal to the law of *G*_0_. We emphasize that the strength of a vertex is given by its label and does not change over time.

**Figure 1.**
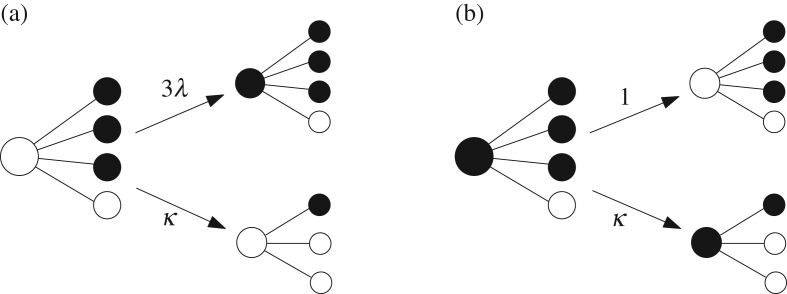
(*a*) The vertex is healthy and has three infected neighbours (represented by black dots) and one healthy one (represented by a white dot, other vertices being not represented). At rate 3λ, the vertex gets infected , while at rate *κ*, it updates and receives a (possibly) completely new set of neighbours. (*b*) The vertex is infected. It turns to healthy at rate 1, and still updates at rate *κ*. Not represented are updates of other vertices that may lead to loss or creation of new neighbours of the vertex.

We consider the contact process, or SIS infection, on this evolving network. Every vertex is in either of two states, infected or healthy. These states evolve according to the following dynamics. Every infected vertex turns to healthy with rate one. Every healthy vertex turns to infected with rate given by the number of infected neighbours, multiplied by a parameter λ>0 called the infection rate. The time evolution of both the network structure and the infection process can be described by a continuous-time Markov chain. Figure 1 illustrates the principal transitions that affect the state of a given vertex or its set of neighbours.

On a finite graph, no matter how the contact process is started, it eventually reaches its unique equilibrium, when all vertices are healthy and the infection has died. We denote by *T*_ext_ the (random) extinction time for the infection. The interesting question in this context is now, once the infection has broken out, how long it takes until the infection becomes extinct. We observe a dichotomy between *slow extinction* on the one hand, when *T*_ext_ is very large, e.g. exponential in the number of vertices, and *fast extinction* on the other, when *T*_ext_ is small, no more than polynomial in the number of vertices. It is shown in [2,17] that the contact process on the static network *G*_0_ exhibits slow extinction for all parameters *γ*,*β*,λ. Our main result shows that this is different for the evolving network model.

**Theorem 2.1.**

*Consider the contact process on the evolving network* (*G*_*t*_)_*t*≥0_, *and assume that, at time t*=0, *every vertex is infected*.

(a) *If* $\gamma >\frac{1}{3}$ (*or equivalently τ*<4), *then whatever the other parameters of the network, there exists a positive constant c such that, uniformly in N*>0:
  $$P(T_{\mathrm{ext}}\leq e^{cN})\leq e^{-cN}.$$
(b) *If* $\gamma <\frac{1}{3}$ (*or equivalently τ*>4), *then there exists a parameter* λ_*c*_>0 *such that, for* λ<λ_*c*_, *there exists a positive constant C such that, uniformly in N*>0, *we have*
  $$E[T_{\mathrm{ext}}]\leq CN^{\gamma }\log N.$$

We say that a family of events $E_{t}$ depending on *t*≥0 holds *exponentially long* if there exists *c*>0 such that, for all *N*:

$$P\left(\underset{s\leq e^{cN}}{⋂}E_{s}\right)\geq 1-e^{-cN}.$$

Using this terminology, we can rephrase (*a*) in theorem 2.1 as saying that in the given regime the infection survives exponentially long.

**Remarks**

(i) The result of (*b*) is true without the assumption on the initial infection. If in (*a*) initially not all vertices are infected, there is additional uncertainty about the outbreak of the infection. In particular, if under the assumptions of (*a*) initially a positive proportion of the vertices are infected, the event *T*_ext_>e^*cN*^ still holds with a positive probability, independent of *N*.
(ii) The result of (*b*) gives the right polynomial order for $E[T_{\mathrm{ext}}]$ when λ<λ_*c*_. Indeed, our study also shows that vertex 1 will be infected at each of its updates, until typically time *N*^*γ*+*o*(1)^. However, it says nothing about the regime λ≥λ_*c*_. It is not hard to see that when λ is *large enough*, the infection survives exponentially long. It would be interesting to show this happens as soon as λ>λ_*c*_, and study the time to extinction when λ=λ_*c*_. This is left for future investigation.

## 3. Comparison with the static model

Before entering the rigorous proof of theorem 2.1, we explain, on a heuristic level, how the time variability of the network can lead to new phenomena that differ both from the rigorous results on static networks, and from the non-rigorous mean-field predictions.

### 3.1. Mean-field predictions

We first describe *informally* the phase transition as predicted by a mean-field calculation. This is based on the principle that instead of taking into account the complex geometry of a network, it suffices to study the behaviour of the contact process around a typical newly infected vertex at a time when the system is locally in a steady state. Such techniques can be quite elaborate and have been widely used and (with correct assumptions in place) typically give correct answers, for example in [19] in the context of adaptive networks.

To demonstrate the difference in behaviour of the static and evolving models, our description focuses on the core assumptions of the prediction and simplifies the actual arguments. The critical infection rate is then the value λ at which such a vertex will, on average, transmit the infection once before its recovery. In the static case, conditionally on the degree *k* of the newly infected vertex, the average number of transmissions before recovery is roughly λ*k*. Assuming that the law of the degree of a newly infected vertex is given by $\tilde{\mu }$, one obtains

$$\lambda_{c}=\frac{1}{\sum k\tilde{\mu }(k)}.$$

We expect the probability of a vertex being infected to be proportional to the number of possible sources of infection, and hence to its degree. Therefore, if the distribution of the degrees of the vertices is given by some probability measure *μ* on N={1,2,…}, the law of the degree of a newly infected vertex is the size-biased distribution $\tilde{\mu }$, defined by

$$\tilde{\mu }(k)=\frac{k\mu (k)}{\sum i\mu (i)},$$

which leads naturally to

$$\lambda_{c}=\frac{1}{\sum k\tilde{\mu }(k)}=\frac{\sum k\mu (k)}{\sum k^{2}\mu (k)},$$

as in [12]. We, therefore, infer that if *μ* is the asymptotic degree distribution of a scale-free graph with power-law exponent *τ*, we get λ_*c*_=0 if *τ*<3 and λ_*c*_>0 if *τ*>3.

The size-biasing effect is crucial in the study of scale-free networks and can also be found in the rigorous analysis of another infection process, the SIR infection, as in [4]. It often leads to a phase transition at the value *τ*=3 [7,26], so that the mean-field predictions loosely described here for the static case did conform to expectations at their initial appearance in [11]. As already mentioned, these mean-field predictions have proved to be inaccurate for the contact process on static scale-free networks. It turns out to be essential for the analysis to consider the *geometry* of the network in the neighbourhood of vertices of large degree, as we explain now.

### 3.2. Slow extinction for the static network

The starting point of the rigorous studies [13,14,17] concerning the contact process on a static scale-free network is the observation that the infection can survive near a vertex of high degree for much longer than predicted by the mean-field calculations. Specifically, Lemma (5.3) in [14] gives an estimate for the survival probability of the contact process on a star graph, which contains only one vertex of degree *k*, and its *k* neighbours, of degree one. It shows that if λ^2^*k* is larger than an absolute constant, then the time to extinction on this star graph is *exponential* in λ^2^*k*. This is owing to *quick reinfections* of the central vertex of degree *k*. More precisely, when the central vertex recovers, it is typically surrounded by many infected vertices that reinfect it almost instantly. This quick reinfection is not picked up by the mean-field calculation above, as it keeps no memory of the actual neighbours of the central vertex.

### 3.3. Heuristic for the evolving network

In our evolving network, we can first control how long the *quick reinfections* can maintain the contact process around a vertex, then make *mean-field-like* calculations rigorous. Consider an infected vertex with large degree *k*. The key observation is that, in the evolving network, quick reinfections alone can maintain the infection around this vertex for a length of time proportional to the degree *k*. In fact, the period of quick reinfection will come to an end if either the vertex updates and then recovers before it has infected its new neighbours, or if the vertex recovers and then updates before its old neighbours have reinfected the vertex. Hence, the infection can be kept in a vertex of degree *k* without external reinfection for λ^2^*k* units of time in the evolving network, which has to be compared with the value one (for mean-field calculations not taking reinfections into account) or $\exp(\lambda^{2}k)$ (for the static network). During the quick reinfection period, the vertex will have infected roughly λ^3^*k*^2^ vertices. After the quick reinfection period the vertex can be infected again, but not by a quick reinfection. It will have to be a *new infection* by a vertex that has become a neighbour before infecting it. For such a new infection, we can rigorously argue that the degree of the newly infected vertex follows the size-biased distribution.

Therefore, if *μ* stands for the asymptotic degree distribution of the scale-free network, we should observe fast extinction roughly if

$$\sum \lambda^{3}k^{2}\tilde{\mu }(k)=\lambda^{3}\frac{\sum k^{3}\mu (k)}{\sum k\mu (k)}<1.$$

Thus, we expect a phase of fast extinction to emerge as soon as $\sum k^{3}\mu (k)<\infty$, which corresponds to *τ*>4 and is in line with (*b*) in theorem 2.1.

This heuristic can be made rigorous and leads formally to lemma 5.2 in the proof of theorem 2.1(*a*). In the proof of theorem 2.1(*b*), the heuristic does not appear explicitly, but has inspired the coupling of proposition 6.1. In this coupling, we only take recoveries into account that are immediately preceded by an update, so that the recovering vertex cannot be quickly reinfected by a neighbour. The update ensures that the memory of the graph topology can be neglected and a suitably defined mean-field approximation can be coupled to the original model in such a way that the extinction time in the approximation is an upper bound for the original extinction time. This coupling idea is the main technical innovation of this paper, and we believe this idea can also be implemented to study other processes on time-varying networks.

## 4. Graphical representation

In this section, we provide an equivalent description of our model by a convenient *graphical representation*. The evolving network model (*G*_*t*_)_*t*≥0_ is represented with the help of the following independent random variables.

(1) For each *x*∈*V* , a Poisson point process of intensity *κ*>0, written $U^{x}={\left(U_{n}^{x}\right)}_{n\geq 1}$, describing the updating times of *x*. We also write $U^{x,y}={\left(U_{n}^{x,y}\right)}_{n\geq 1}$ for the union $U^{x}\cup U^{y}$, which is a Poisson point process of intensity 2*κ*, describing the updating times of the potential edge {*x*,*y*}⊂*V* .
(2) For each potential edge {*x*,*y*}⊂*V* , a sequence of independent random variables ${\left(C_{n}^{x,y}\right)}_{n\geq 0}$, all Bernoulli with parameter *p*_*x*,*y*_, describing the presence/absence of the edge in the network after the successive updating times of the potential edge {*x*,*y*}. More precisely, if *t*≥0, then {*x*,*y*} is an edge at time *t* if and only if $C_{n}^{x,y}=1$ for $n=|[0,t]\cap U^{x,y}|$.

Given the network, we represent the infection by means of the following set of independent random variables.

(3) For each *x*∈*V* , a Poisson point process of intensity one, written $R^{x}={\left(R_{n}^{x}\right)}_{n\geq 1}$, describing the recovery times of *x*.
(4) For each {*x*,*y*}⊂*V* , a Poisson point process $I_{0}^{x,y}$ with intensity λ describing the infection times along the edge {*x*,*y*}. Only the trace $I^{x,y}$ of this process on the set
  $$\overset{\infty }{\underset{n=0}{⋃}}\{[U_{n}^{x,y},U_{n+1}^{x,y}):C_{n}^{x,y}=1\}\subset [0,\infty )$$
  can actually cause infections. Write ${\left(I_{n}^{x,y}\right)}_{n\geq 1}$ for the ordered points of $I^{x,y}$. If at time $I_{n}^{x,y}$ vertex *x* is infected and *y* is healthy, then *x* infects *y*. If *y* is infected and *x* is healthy, then *y* infects *x*. Otherwise, nothing happens.

The infection is now described by a process (*X*_*t*_(*x*))_*x*∈*V*_ with values in {0,1}^*V*^, such that *X*_*t*_(*x*)=1 if *x* is infected at time *t*, and *X*_*t*_(*x*)=0 if *x* is healthy at time *t*. Formally, the infection process *X*_*t*_(*x*) associated to this graphical representation and to a starting set *A*_0_ of infected vertices is the càdlàg process with *X*_0_=1_*A*_0__ evolving only at times $t\in R^{x}\cup \overset{\infty }{\underset{n=1}{⋃}}I_{n}^{x,y}$, according to the following rules:

— If $t\in R^{x}$, then *X*_*t*_(*x*)=0 (whatever *X*_*t*−_(*x*)),
— If $t\in I^{x,y}$, then
  $$(X_{t}(x),X_{t}(y))=\left\{ \begin{array}{ll} \left(0,0\right) & \text{if }(X_{t-}(x),X_{t-}(y))=(0,0),\\ \left(1,1\right) & \text{otherwise.} \end{array}\right.$$

## 5. Proof of theorem 2.1(*a*): exponential extinction time

Fix $\gamma >\frac{1}{3}$ and note that it suffices to look at small values of the infection rate λ>0. Our strategy is to focus on highly connected vertices, which we call *stars*. Informally, when a star is infected, typically a proportion of its neighbours is infected at any time. When the star recovers, it is likely to be quickly reinfected by its neighbours, keeping the infection alive for a long time. Only when the star updates before it is reinfected by its neighbours, the recovery is sustained. We use a coupling to look at infections between stars, taking into account only the sustained recoveries.

More precisely, we partition the vertex set *V* into two sets:

$$S:=\{1,\ldots ,\lfloor \lambda^{(2+\alpha )/\gamma }N\rfloor \}\;\text{and}\;C:=\{\lfloor \lambda^{(2+\alpha )/\gamma }N\rfloor +1,\ldots ,N\},$$

where *α*>0 is a constant. We call the vertices in S *stars*, those in C *connectors*. Note that a star has an average degree of order λ^−2−*α*^, which is large when λ is small. The constant *α*>0 is chosen large enough so that we have

5.1 $$\left(\frac{1}{\gamma }-3\right)\alpha +\frac{2}{\gamma }-3<0,$$

which is possible since $\gamma >\frac{1}{3}$. The reason for this choice will become clear only at the end of the proof. If *x* and *y* are stars, then

$$p_{x,y}\geq {\tilde{p}}_{x,y}:=\beta \lambda^{-4-2\alpha }N^{-1},$$

while if *x* is a star and *y* a connector, then

$$p_{x,y}\geq {\tilde{p}}_{x,y}:=\beta \lambda^{-2-\alpha }N^{-1}.$$

By a simple coupling argument, it is enough to prove (*a*) on the modified network where the connection probabilities are replaced by ${\tilde{p}}_{x,y}$ and there are no connections between connectors. We denote this process by $({\tilde{X}}_{t}{\left(x)\right)}_{x\in V}$.

### 5.1. Connector update and recovery times

For the infection to survive for a long time around a star, it requires connectors which can get infected and then reinfect the star. The first lemma ensures that, at any time, there are sufficiently many connectors that do not update or recover too quickly. For any t∈N∪{0} let

$$C_{t}:=\{y\in C:(R^{y}\cup U^{y})\cap [t,t+2]=\emptyset \}.$$

**Lemma 5.1.**

*For any sufficiently small η*>0, *the event* $E_{t}:=\{|C_{t}|\geq \eta N\}$ *holds exponentially long*.

For y∈C, the events $\left\{(R^{y}\cup U^{y})\cap [s,s+1]=\emptyset \right\}$ are independent with a probability bounded from zero. Therefore, there exist *η*,*c*′>0 not depending on *s* such that P(|{y∈C: $(R^{y}\cup U^{y})\cap [s,s+1]=\emptyset \}|<\eta N)\leq e^{-c^{\prime }N}$. Picking 0<*c*<*c*′, we get that

$$P\left(\underset{s\leq e^{c}N}{⋃}\{|C_{s}|<\eta N\}\right)\leq \sum_{s=1}^{\left\lceil e^{cN}\right\rceil }P(|\{y\in C:(R^{y}\cup U^{y})\cap [s,s+1]=\emptyset \}|<\eta N)\leq e^{(c-c^{\prime })N},$$

and passing to the complementary events completes the proof of lemma 5.1.

From now on, we work conditionally on the realization of the recovery and update times of the connectors, ${\left(R^{y}\right)}_{y\in C}$ and ${\left(U^{y}\right)}_{y\in C}$, and assume that $E_{t}$ holds exponentially long, without mentioning explicitly the implied constants.

### 5.2. Extinction time for one star

Let *T*_λ_:=λ^−*α*′^, where *α*′ is a positive constant strictly smaller than *α*, such that

5.2 $$\left(\frac{1}{\gamma }-2\right)\alpha -\alpha^{\prime }+\frac{2}{\gamma }-3<0,$$

which is possible by (5.1). We shall see that a star, alone with its neighbouring connectors, is likely to sustain the infection up to time of order *T*_λ_. Note that when λ is small, *T*_λ_ becomes larger, but λ^−2^*T*_λ_ stays negligible compared with the average degree of a star, which is of order λ^−2−*α*^.

For each star *x*, we now define a process *X*^*x*^ called the *infection process around *x**. It is the infection process on the graph with vertex set {x}∪C and edges in {x}×C evolving as in the graphical representation from the data of the original graph, with initial condition

$$X_{0}^{x}(y)=\left\{ \begin{array}{ll} 0 & \text{if }y\neq x,\\ X_{0}(x) & \text{if }y=x. \end{array}\right.$$

The data of the original graph are the fixed recovery and update times of the connectors, ${\left(R^{y}\right)}_{y\in C}$ and ${\left(U^{y}\right)}_{y\in C}$ as well as the recovery times $R^{x}$, updating times $U^{x}$, Bernoulli variables ${\left(C_{n}^{x,y}\right)}_{n}$ with parameter ${\tilde{p}}_{x,y}$, and infection times $I_{0}^{x,y}$, for y∈C. It is important to note that, for distinct x,w∈S and y∈C, the state of $X_{t}^{x}(y)$ is not affected by infections from *w* and may well be different from $X_{t}^{w}(y)$. However, the infection process $\tilde{X}$ satisfies

$${\tilde{X}}_{t}(x)\geq X_{t}^{x}(x)\;\text{for all }t\geq 0,x\in S,$$

as the additional edges in the original graph can only lead to more vertices being infected. Given the realizations of ${\left(R^{y}\right)}_{y\in C}$ and ${\left(U^{y}\right)}_{y\in C}$, the process *X*^*x*^ depends only on the updating times and recovery times at *x*, and the infection times on edges {*x*,*y*} for y∈C. Therefore, *X*^*x*^ and *X*^*w*^ are conditionally independent. We say the infection *persists on [0,*T*]* at a star x∈S if

— $X_{T}^{x}(x)=1$ and
— $|\{t\in U^{x}\cap [0,T-1]:X_{t}^{x}(x)=1,(U^{x}\cup R^{x})\cap [t,t+1]=\{t\}\}|\geq \frac{1}{2}\kappa \;e^{-(1+\kappa )}T.$

The second condition ensures that there are enough updating times after which *x* stays infected for a unit of time without any update or recovery. This will be useful later to control inter-infections of stars.

**Lemma 5.2.**

*Let* x∈S *be a star and* $P_{x}$ *be the event that the infection persists on* [0,*T*_λ_] *at x*. *Then*:

$$\lim_{\lambda ↓0}\underset{N\to \infty }{lim inf}P(P_{x}|{\tilde{X}}_{0}(x)=1)=1.$$

We say an event depending on λ and *N* holds *with high probability* if its probability is arbitrarily close to one when first λ is chosen small enough, and then *N* is sufficiently large. In this sense, lemma 5.2, which we prove in the remainder of this section, states that on [0,*T*_λ_] persistence holds with high probability. A first simple observation is that, with high probability, we have $|U^{x}\cap [0,T_{\lambda }]|\leq 2\kappa T_{\lambda }$ and

$$\begin{array}{r} |\{t\in U^{x}\cap [0,T_{\lambda }-1]:(U^{x}\cup R^{x})\cap [t,t+1]=\{t\}\}|\geq \frac{1}{2}\kappa \;e^{-(1+\kappa )}T_{\lambda }. \end{array}$$

It remains to prove that we also have, with high probability, that $X_{U_{n}^{x}}^{x}(x)=1$ for *n*≤2*κT*_λ_. Set *U*′_0_=0 and recursively $U_{n+1}^{\prime }=\min\{1+U_{n}^{\prime },U^{x}\cap (U_{n}^{\prime },\infty )\}$, so that all time intervals [*U*′_*n*_,*U*′_*n*+1_] have length at most one. It is enough to prove that, with high probability, we have $X_{U_{n}^{\prime }}^{x}(x)=1$ for *n*≤(1+2*κ*)*T*_λ_. For *t*≤*T*_λ_, define

$$C_{t}(x):=\{y\in C_{\left\lfloor t\right\rfloor }:\{x,y\}\in G_{t}\},$$

the set of neighbours of *x* that are in $C_{\left\lfloor t\right\rfloor }$. These are neighbours that we will use to help maintain the infection in *x*. The following lemma shows that, with high probability, there are enough of them at any time in [0,*T*_λ_].

**Lemma 5.3.**

$$\lim_{\lambda ↓0}\underset{N\to \infty }{lim inf}P(|C_{U_{n}^{\prime }}(x)|\geq \frac{1}{2}\eta \beta \lambda^{-2-\alpha }\;\forall n\leq \lfloor (1+2\kappa )T_{\lambda }\rfloor )=1.$$

The result of lemma 5.3 follows if $P\{|C_{U_{n}^{\prime }}(x)|<\frac{1}{2}\eta \beta \lambda^{-2-\alpha }\}$ is, for large enough *N*, uniformly in *n*, bounded by a function that is negligible compared with λ^*α*′^. To this end, observe that, conditionally on *U*′_*n*_, the cardinality of $C_{U_{n}^{\prime }}(x)$ is binomial with parameters $\left|C_{\left\lfloor U_{n}^{\prime }\right\rfloor }\right|$ and *βλ*^−2−*α*^*N*^−1^. As we work on the event $\left\{|C_{\left\lfloor U_{n}^{\prime }\right\rfloor }|\geq \eta N\right\}$, the cardinality of $C_{U_{n}^{\prime }}(x)$ is asymptotically bounded from below by a Poisson variable with parameter *ηβλ*^−2−*α*^. By a standard large deviation bound, the probability that it is less than $\frac{1}{2}\eta \beta \lambda^{-2-\alpha }$ decays exponentially fast in λ^−2−*α*^, completing the proof.

Using lemma 5.3, we can work conditionally on the event

5.3 $$\{|C_{U_{n}^{\prime }}(x)|\geq \frac{1}{2}\eta \beta \lambda^{-2-\alpha }\forall n\leq \lfloor (1+2\kappa )T_{\lambda }\rfloor \}.$$

Lemma 5.2 is proved once we prove the following lemma.

**Lemma 5.4.**

*Uniformly for all* 0≤*n*≤⌊(1+2*κ*)*T*_λ_⌋, *we have*

$$P(X_{U_{n+1}^{\prime }}^{x}(x)=0|X_{U_{n}^{\prime }}^{x}(x)=1)=o(\lambda^{\alpha^{\prime }}),$$

*where the probability is conditional on* (*5.3*).

The probability on the left does not depend on *n*, so we may take *n*=0 and $X_{0}^{x}(x)=1$. On the event $\left\{X_{U_{1}^{\prime }}^{x}(x)=0\right\}$, we necessarily have $R^{x}\cap (0,U_{1}^{\prime })\neq \emptyset .$ Define random variables

$$R=\inf R^{x}\cap (0,U_{1}^{\prime }),\;L=U_{1}^{\prime }-\sup R^{x}\cap (0,U_{1}^{\prime }).$$

The strategy is to find a connector *y* that gets infected by *x* during the time interval [0,*R*], and then reinfects *x* on [*U*′_1_−*L*,*U*′_1_]. We look for such a connector in the set $C_{0}(x)$, as these are connected to *x* during the whole interval [0,*U*′_1_], and do not recover during this time. For a given $y\in C_{0}(x)$, the probability that the back-and-forth infection between *x* and *y* occurs is

$$(1-e^{-\lambda R})(1-e^{-\lambda L})\geq \frac{1}{4}\lambda^{2}LR,$$

where the bound uses the inequality 1−e^−*x*^≥*x*/2 for *x*∈[0,1] and the fact that *L*,*R*<1. Hence, the probability that this fails for all $y\in C_{0}(x)$ is bounded by

$${\left(1-\frac{\lambda^{2}LR}{4}\right)}^{\left|C_{0}(x)\right|}\leq \exp\left(\frac{1}{2}\eta \beta \lambda^{-2-\alpha }\log\left(1-\frac{1}{4}\lambda^{2}LR\right)\right).$$

On the event that *LR*≥λ^*α*′′^, with $\alpha^{\prime \prime }=\frac{1}{2}(\alpha +\alpha^{\prime })$, the bound is *o*(λ^*α*′^), as requested. We conclude the proof showing that the probability that *LR* is smaller than λ^*α*′′^ is $O(\lambda^{\alpha \prime \prime }\log(\lambda^{-1}))$. To prove this, write *L*≥*L*′∧*L*′′ with $L^{\prime }=1-\sup(R^{x}\cap (0,1))$ and $L^{\prime \prime }=U_{1}^{x}-\sup(R^{x}\cap (0,U_{1}^{x}))$, and bound separately $P(L^{\prime }R\leq \lambda^{\alpha \prime \prime })$ and $P(L^{\prime \prime }R\leq \lambda^{\alpha \prime \prime }).$

For the first term, we bound separately the probability of the three events {*L*′≤2λ^*α*′′^}, {*R*≤2λ^*α*′′^} and $\left\{L^{\prime }\leq \frac{1}{2},R\leq \frac{1}{2},L^{\prime }R\leq \lambda^{\alpha \prime \prime }\right\}$. The probability of the first two events is easily seen to be of order *O*(λ^*α*′′^). On $\left\{L^{\prime }\leq \frac{1}{2},R\leq \frac{1}{2}\right\}$, the random variables *L*′ and *R* are independent and their laws have bounded density on $\left[0,\frac{1}{2}\right]$. Hence, the probability of the third event is bounded by a constant multiple of

$$\int_{0}^{1/2}dx\int_{0}^{1/2}dy1\{xy\leq \lambda^{\alpha \prime \prime }\}=\int_{0}^{1/2}dx\left(\frac{\lambda^{\alpha \prime \prime }}{x}\wedge \frac{1}{2}\right)=O(\lambda^{\alpha \prime \prime }\log(\lambda^{-1})).$$

Finally, the bound on $P(L^{\prime \prime }R\leq \lambda^{\alpha \prime \prime })$ is similar and easier, using that *L*′′ and *R* are independent.

### 5.3. Reinfections between stars

Consider an initial condition where the stars in D⊂S are infected, and those in S∖D are healthy. From the preceding subsection, we know that, independently for each infected star, the infection is likely to persist at that star. Denote by $D^{\prime }$ the set of infected stars at which the infection persists on [0,*T*_λ_]. In this subsection, we prove a quantitative lemma stating that each healthy star has independently some probability of becoming infected by some star $s\in D^{\prime }$, and then be infected at time *T*_λ_. We condition on the independent infection processes ${\left(X^{x}\right)}_{x\in D}$, which in particular determine $D^{\prime }$. For x∈S∖D, we define

$$T_{x}:=\inf\{t>0:\exists y\in D^{\prime },\;X_{t}^{y}(y)=1,\;t\in I^{x,y}\}.$$

Observe that if *T*_*x*_ is finite, then *x* is necessarily infected at this time, namely we have ${\tilde{X}}_{T_{x}}(x)=1$. If *T*_*x*_≤*T*_λ_, we define an infection process similar to the process *X*^*x*^, but on the restricted time interval [*T*_*x*_,*T*_λ_]. More precisely, define the process ${\left({\tilde{X}}_{t}^{x}\right)}_{t\in [T_{x},T_{\lambda }]}$ as the infection process on the graph with vertex set {x}∪C and edges in {x}×C evolving as in the graphical representation from the data of the original graph, with initial condition

$${\tilde{X}}_{T_{x}}^{x}(y)=\left\{ \begin{array}{ll} 0 & \text{if }y\neq x,\\ 1 & \text{if }y=x. \end{array}\right.$$

We further extend this process to [0,*T*_λ_] by letting ${\tilde{X}}_{t}^{x}(y)=0$ if *t*<*T*_*x*_ (so the process is well-defined even if *T*_*x*_>*T*_λ_). As in the definition of *X*^*x*^, we note that the processes ${\tilde{X}}^{x}$, for x∈S∖D, are independent conditionally on ${\left(X^{x}\right)}_{x\in D}$. Moreover, for all x∈S∖D we have

$${\tilde{X}}_{T_{\lambda }}(x)\geq {\tilde{X}}_{T_{\lambda }}^{x}(x),$$

as $\tilde{X}$ is defined on a graph with more edges and initial infections than ${\tilde{X}}^{x}$. By a slight variation of lemma 5.2 we see that, conditionally on *T*_*x*_<*T*_λ_, the probability that *x* is infected at time *T*_λ_ goes to 1 when λ goes to 0 and *N* is large. Therefore, if λ is small and *N* large enough, every healthy star has independently probability at least

$$\frac{1}{2}P(T_{x}<T_{\lambda }|{\left(X^{y}\right)}_{y\in D}),$$

of being infected at time *T*_λ_. The following lemma bounds this probability from below by a quantity depending on $\left|D^{\prime }\right|$.

**Lemma 5.5.**

If x∈S∖D is a healthy star, i.e. ${\tilde{X}}_{0}(x)=0$, then

$$P(T_{x}<T_{\lambda }|{\left(X^{y}\right)}_{y\in D})\geq 1-\exp\left(-\frac{1}{4}\beta \kappa \;e^{-(1+\kappa )}\frac{|D^{\prime }|\lambda^{-3-2\alpha }T}{N}\right).$$

Condition on ${\left(X^{y}\right)}_{y\in D}$, the infection processes around each y∈D. We first look at an individual star *y*≠*x* and suppose that the infection persists at *y*. We further condition on the updating times $U^{x}$, and hence on the updating times of the edge {*x*,*y*}, namely ${\left(U_{n}^{x,y}\right)}_{n\geq 0}$. For *n*≥0, write

$$t_{n}:=\int_{\left[U_{n}^{x,y},U_{n+1}^{x,y}\wedge T_{\lambda }\right]}1\{X_{t}^{y}(y)=1\}\;dt,$$

which is a lower bound on the total time when vertex *y* is infected between consecutive updating times. The probability that *y* does not infect *x* is therefore bounded from above by

$$\begin{array}{rl} \prod_{n\geq 0}(1-{\tilde{p}}_{x,y}(1-e^{-\lambda t_{n}})) & \leq \prod_{n\geq 0}\left(1-\lambda {\tilde{p}}_{x,y}\frac{1}{2}\min\{1,t_{n}\}\right)\\ & \leq \exp\left(-\frac{1}{2}({\tilde{p}}_{x,y}\lambda )\sum_{n\geq 0}\min\{1,t_{n}\}\right). \end{array}$$

Using the lower bound for the sum we get from the fact that the infection persists around *y*, we continue the inequality by

∏n≥0(1−p~x,y(1−e−λtn))≤exp(−14κ e−(1+κ)λp~x,yTλ)≤exp(−14βκ e−(1+κ)λ−3−2αTλN).

Recalling the independence of the infection processes *X*^*y*^ for different stars y∈S, we see that this upper bound corresponds to a coupling of the events that *y* does not infect *x*, for $y\in D^{\prime }$, that are conditionally independent given the realization of $U^{x}$. Hence, the probability that no vertex $y\in D^{\prime }$ infects *x* is bounded by the right hand side to the power $\left|D^{\prime }\right|$, and the result follows.

### 5.4. Discrete infection process

We study the infection process on stars at discrete times 0,*T*_λ_,2*T*_λ_,… For *n*≥0, we denote by $D_{n}$ the set of stars x∈S with ${\tilde{X}}_{nT_{\lambda }}(x)=1$, and by $A_{n}$ the set of stars $x\in D_{n}$ such that the infection persists on [*nT*_λ_,(*n*+1)*T*_λ_] at *x*. We start with $D_{0}=S$, i.e. initially all stars are infected.

By lemma 5.2, the cardinality of $A_{0}$ is stochastically larger than a binomial random variable with parameters |S| and $\frac{1}{2}$ if λ is small and *N* large. More generally, for *n*≥0, the cardinality of $A_{n}$ conditionally on $\left|A_{0}|,\ldots ,|A_{n-1}\right|$ and on $\left|D_{0}|,\ldots ,|D_{n}\right|$ is stochastically larger than a binomial random variable with parameters $\left|D_{n}\right|$ and $\frac{1}{2}$. By lemma 5.5, conditionally on $\left|A_{0}|,\ldots ,|A_{n}\right|$ and on $\left|D_{0}|,\ldots ,|D_{n}\right|$, the cardinality of $D_{n+1}\setminus A_{n}$ is stochastically larger than a binomial random variable with parameters $\left|S|-|D_{n}\right|$ and

5.4 $$\frac{1}{2}\left(1-\exp\left(-\frac{1}{4}\beta \kappa \;e^{-(1+\kappa )}\frac{|A_{n}|\lambda^{-3-2\alpha }T_{\lambda }}{N}\right)\right).$$

Next, we show inductively that, with high probability, the event $E_{n}:=\{|D_{n}|\geq \frac{1}{4}\;|S|\}$ holds exponentially long. Let *n*≥0 and condition on $E_{n}$. Then, as the number of stars is linear in *N* and each persists on [*nT*_λ_,(*n*+1)*T*_λ_] independently with probability at least $\frac{1}{2}$, we infer that the event $\left|A_{n}|\geq \frac{2}{5}|D_{n}|\geq \frac{1}{10}|S\right|$ holds with probability at least 1−e^−*cN*^ for some *c*>0, which depends on λ but not on *N*. If this holds, then we have

$$\frac{|A_{n}|\lambda^{-3-2\alpha }T_{\lambda }}{N}\geq \frac{1}{10}\lambda^{(1/\gamma -2)\alpha -\alpha^{\prime }+2/\gamma -3}.$$

The exponent in λ is negative, by our choice of *α* and *α*′; see (5.2). Therefore, choosing λ smaller if necessary, we can ensure that the value of (5.4) is larger than $\frac{1}{3}$, which means that each non-infected star, or vertex in $S\setminus D_{n}$, has independently probability at least $\frac{1}{3}$ to get infected. This, together with $\left|A_{n}|\geq \frac{2}{5}|D_{n}\right|$, ensures that the event $\left|D_{n+1}|\geq \frac{1}{4}|S\right|$ holds with probability at least 1−e^−*cN*^ for some (new) constant *c*. Altogether, we have proved that the infection persists exponentially long.

## 6. Proof of theorem 2.1(*b*): polynomial extinction time

In this section, we propose a coupling of the contact process on an evolving scale-free network, with a *mean-field infection model*. In the mean-field infection model, an infected vertex recovers only when for the original process we can ensure that the recovery is sustained and not subject to a quick reinfection. This is the case when the recovery is immediately preceded by an update of the vertex, so that it is no longer exposed to its original infected neighbours. This allows us to neglect information on the graph structure around the infected vertex prior to the update. Hence, we can set up the mean-field infection model independently of the topology of the evolving network, namely as an infection process on the complete graph with appropriately decreased infection rates. Finally, the mean-field infection model can be studied with standard techniques, and we can show that the time to extinction is polynomial when $\gamma <\frac{1}{3}$.

### 6.1. Mean-field infection model

To setup the mean-field infection model, for every *x*∈*V* , we use the point processes $R^{x}$ and $U^{x}$ introduced for graphical representation of the original infection process. In addition, for each potential edge {*x*,*y*}⊂*V* , we define an independent Poisson point process $J^{x,y}={\left(J_{n}^{x,y}\right)}_{n\geq 1}$ with intensity λ*p*_*x*,*y*_. The sets $J^{x,y}$ describe infection times for this process, but the sets $R^{x}$ and $U^{x}$ now have a different meaning. They describe how recoveries can happen, but in a more involved way. To describe the infection, we use a Markov process (*Y*_*t*_(*x*))_*x*∈*V*_ with values in {0,1,2}^*V*^, where a vertex *x* can take three different values, the value 0 still meaning *healthy*, the value 2 meaning *infected* and the value 1 meaning infected but *ready* to recover. The process evolves according to the following rules:

(1) if $t\in J^{x,y}$, then
  $$(Y_{t}(x),Y_{t}(y))=\left\{ \begin{array}{ll} \left(0,0\right) & \text{if }(Y_{t-}(x),Y_{t-}(y))=(0,0),\\ \left(2,2\right) & \text{otherwise,} \end{array}\right.$$
(2) if $t\in U^{x}$, then
  $$Y_{t}(x)=\left\{ \begin{array}{ll} 1 & \text{if }Y_{t-}(x)=2,\\ Y_{t-}(x) & \text{otherwise,} \end{array}\right.$$
(3) if $t\in R^{x}$, then
  $$Y_{t}(x)=\left\{ \begin{array}{ll} 0 & \text{if }Y_{t-}(x)=1,\\ Y_{t-}(x) & \text{otherwise.} \end{array}\right.$$

In other words, once a vertex *x* is infected, in order to recover, it has to observe an ‘updating’ time in $U^{x}$, then a ‘recovery’ time in $R^{x}$ and no infection time in between (in $\underset{y}{⋃}J^{x,y}$).

### 6.2. Coupling

The next proposition states that if we fix the initial conditions of the infection processes *X* and *Y* so that *X*_0_≤*Y*_0_, then there exists a coupling maintaining the ordering *X*_*t*_≤*Y*_*t*_ for all times.

**Proposition 6.1.**

*Fix deterministic initial conditions X*_0_≤*Y*_0_. *One can construct, on the same probability space, the evolving network* (*G*_*t*_)_*t*≥0_, *the infection process* (*X*_*t*_(*x*))_*x*∈*V*,*t*≥0_ *on this network and the mean-field infection process* (*Y*_*t*_(*x*))_*x*∈*V*,*t*≥0_, *such that X*_*t*_(*x*)≤*Y*_*t*_(*x*) *for all x*∈*V and t*≥0.

We stress that the coupling uses the knowledge of the initial condition *X*_0_. As indicated above, we choose the same $U^{x}$ and $R^{x}$ for both models. The coupling will actually be a coupling between the processes $J^{x,y}$ and the processes $I^{x,y}$, which are determined as before by variables $I_{0}^{x,y}$, $C_{n}^{x,y}$ and $U^{x,y}=U^{x}\cup U^{y}$. Given the latter, the set $J^{x,y}$ is defined as a subset of $I_{0}^{x,y}$, obtained by percolation of parameter *p*_*x*,*y*_, and hence is a Poisson point process of parameter λ*p*_*x*,*y*_, as requested. However, this percolation procedure will be dependent on the process *X* and on the $C_{n}^{x,y}$, as we now explain. We introduce the following notions:

— an *infection time* is an infection time of the original process, namely a time *t*≥0 belonging to some $I^{x,y}$, or equivalently belonging to some $I_{0}^{x,y}$ and such that $C_{n}^{x,y}=1$ if $t\in (U_{n}^{x,y},U_{n+1}^{x,y})$;
— a *potential infection time* is any time *t* in some $I_{0}^{x,y}$;
— a *true infection time* is an infection time *t* satisfying the additional condition $\max\{X_{t-}(x),X_{t-}(y)\}=1$; and
— a *potential true infection time* is a potential infection time *t* satisfying additionally $\max\{X_{t-}(x),X_{t-}(y)\}=1$.

It is clear that only true infection times can play any role in the spread of the infection. Observe also that the notion of true infection times depends monotonically on the infection process up to time *t*−, in the sense that any true infection time for *X* would also be a true infection time for a process *X*′≥*X*. For each *x*,*y* and *n*, we denote by $F_{n}^{x,y}$ the first potential true infection time in $\left(U_{n}^{x,y},U_{n+1}^{x,y}\right)$ if there is any:

$$F_{n}^{x,y}=\inf\{t\in I_{0}^{x,y}\cap (U_{n}^{x,y},U_{n+1}^{x,y}):\max\{X_{t-}(x),X_{t}-(y)\}=1\},$$

with the convention inf∅=+∞. The important observation now is that the time $F_{n}^{x,y}$ and the infection process strictly before that time, on $\left[0,F_{n}^{x,y}\right)$, are independent of $C_{n}^{x,y}$. This follows from the fact that, on $\left(U_{n}^{x,y},U_{n+1}^{x,y}\right)$, before the first potential true infection, there cannot be any true infection along *x*,*y*, whatever the value of $C_{n}^{x,y}$, and thus the infection process can be determined independently of $C_{n}^{x,y}$.

To construct $J^{x,y}$ let $t\in I_{0}^{x,y}\cap (U_{n}^{x,y},U_{n+1}^{x,y})$. If *t* is the first potential true infection, i.e. if $t=F_{n}^{x,y}$, we let $t\in J^{x,y}$ if $C_{n}^{x,y}=1$ and $t\notin J^{x,y}$ if $C_{n}^{x,y}=0$. Otherwise, sample an independent Bernoulli *p*_*x*,*y*_ random variable to determine whether *t* is kept in $J^{x,y}$ or not.

It is clear that $J^{x,y}$ has the required law, i.e. it is a Poisson point process of intensity λ*p*_*x*,*y*_ obtained by percolation of parameter *p*_*x*,*y*_ on $I_{0}^{x,y}$. We now explain why the mean-field process *Y* has to stay above *X* if it has started with *Y*_0_≥*X*_0_. To this end, we have to ensure two things:

— if a vertex *x* recovers for the process *Y* at some time *t*, then it also recovers for the process *X* (if it was infected); and
— if $t\in I^{x,y}\cap (U_{n}^{x,y},U_{n+1}^{x,y})$ is a true infection time for the process *X*, then we have (*Y*_*t*_(*x*),*Y*_*t*_(*y*))≥(1,1).

The first item is obvious as *Y* recovers only at times $t\in R^{x}$, and *X* also recovers at these times. To verify the second item, observe that under our assumption we have $C_{n}^{x,y}=1$, and hence on $\left(U_{n}^{x,y},U_{n+1}^{x,y}\right)$ potential infections, resp. potential true infections, coincide with infections, resp. true infections.

Now we distinguish two cases. In the first case, $t=F_{n}^{x,y}$ is the first true infection time for the process *X* on $\left(U_{n}^{x,y},U_{n+1}^{x,y}\right)$. By checking all the potential infection times $t\in I_{0}^{x,y}$ successively, we ensure that *Y* is above *X* up to time *t*−. As $t\in J^{x,y}$ by construction, we obtain (*Y*_*t*_(*x*),*Y*_*t*_(*y*))=(2,2)≥(1,1). In the second case, *t* is not the first true infection time on $\left(U_{n}^{x,y},U_{n+1}^{x,y}\right)$. Then we have $F_{n}^{x,y}<t$, and at time $F_{n}^{x,y}\in J^{x,y}$, we have set the value of *Y* in *x* and *y* to be 2. These values cannot decrease before the next updating time for *x* or *y*, namely $U_{n+1}^{x,y}$. We deduce again *Y*_*t*_(*x*)=*Y*_*t*_(*y*)=2, and this completes the proof.

### 6.3. Study of the mean-field infection model

In this part, we show that, for sufficiently small λ>0, the infection dies in polynomial time for the mean-field model. We use a simple supermartingale technique, by introducing a process *M*(*t*) defined by

$$M(t):=\sum_{x=1}^{N}1_{\left\{Y_{t}(x)=2\right\}}s_{2}(x)+\sum_{x=1}^{N}1_{\left\{Y_{t}(x)=1\right\}}s_{1}(x).$$

The values *s*_1_(*x*) and *s*_2_(*x*), for *x*∈{1,…,*N*}, are non-negative scores, associated to infected vertices, which should roughly quantify to which extent the fact that *x* is infected contributes to the survival of the infection. Actually, we want to define them in such a way that, if the infection rate λ is small, *M* is a tractable supermartingale with respect to the filtration $\left(F_{t}\right)$ generated by (*Y*_*t*_). Informally, *s*_2_(*x*) should be larger than *s*_1_(*x*), and, for *i*=1,2, we should have *s*_*i*_(*x*) larger than *s*_*i*_(*x*′) if *x*<*x*′, as *p*_*x*,*y*_≥*p*_*x*′,*y*_ for all *y*≠*x*,*x*′. Applying the formal generator of the mean-field model yields

$$\begin{array}{rl} \frac{1}{dt}E[M(t+dt)-M(t)|F_{t}] & =\sum_{x:Y_{t}(x)=2}\kappa (s_{1}(x)-s_{2}(x))\\ & \;+\sum_{x:Y_{t}(x)=1}\left(-s_{1}(x)+\sum_{y:y\neq x}\lambda p_{x,y}(s_{2}(x)-s_{1}(x))\right)\\ & \;+\sum_{x:Y_{t}(x)=0}\left(\sum_{\begin{array}{c} y:y\neq x, \end{array}Y_{t}(y)\geq 1}\lambda p_{x,y}s_{2}(x)\right). \end{array}$$

In this equality, we have written, for each vertex *x*, the change of its contribution to the total score, namely *s*_1_(*x*)1{*Y*_*t*_(*x*)=1}+*s*_2_(*x*)1{*Y*_*t*_(*x*)=2}, induced by the infinitesimal transition probabilities of the infection process changing the state of *x*. The third sum can be rewritten as

$$\sum_{x:Y_{t}(x)\geq 1}\sum_{\begin{array}{c} y:y\neq x,\\ Y_{t}(y)=0 \end{array}}\lambda p_{x,y}s_{2}(y)\leq \sum_{x:Y_{t}(x)\geq 1}\sum_{y}\lambda p_{x,y}s_{2}(y).$$

Writing $S(x):=\sum_{y}p_{x,y}$ and $T(x):=\sum_{y}p_{x,y}s_{2}(y),$ we get the following bound:

$$\begin{array}{rl} \frac{1}{dt}E[M(t+dt)-M(t)|F_{t}] & \leq \sum_{x:Y_{t}(x)=2}\lambda T(x)-\kappa (s_{2}(x)-s_{1}(x))\\ & \;+\sum_{x:Y_{t}(x)=1}\lambda T(x)+\lambda S(x)(s_{2}(x)-s_{1}(x))-s_{1}(x). \end{array}$$

It is easy to bound *S*(*x*), which is the average degree of *x*, as

$$S(x)\leq \frac{\beta }{N^{1-2\gamma }x^{\gamma }}\sum_{y=1}^{N}y^{-\gamma }\leq \frac{\beta }{1-\gamma }{\left(\frac{N}{x}\right)}^{\gamma }.$$

We now choose *s*_1_ and *s*_2_ as

$$s_{1}(x)={\left(\frac{N}{x}\right)}^{2\gamma }\;s_{2}(x)=s_{1}(x)+{\left(\frac{N}{x}\right)}^{\gamma }.$$

Then, we can bound *T*(*x*) as

$$T(x)\leq \frac{2\beta }{N^{1-4\gamma }x^{\gamma }}\sum_{y=1}^{N}y^{-3\gamma }\leq \frac{2\beta }{1-3\gamma }{\left(\frac{N}{x}\right)}^{\gamma },$$

where we used $\gamma <\frac{1}{3}$. Therefore, for any *x*, we have

$$\lambda T(x)-\kappa (s_{2}(x)-s_{1}(x))\leq \left(\frac{2\lambda \beta }{1-3\gamma }-\kappa \right){\left(\frac{N}{x}\right)}^{\gamma }$$

and

$$\lambda T(x)+\lambda S(x)(s_{2}(x)-s_{1}(x))-s_{1}(x)\leq \frac{2\lambda \beta }{1-3\gamma }{\left(\frac{N}{x}\right)}^{\gamma }+\left(\frac{\lambda \beta }{1-\gamma }-1\right){\left(\frac{N}{x}\right)}^{2\gamma }.$$

If λ is small enough, the negative terms compensate all the positive ones, and the first of the above terms is bounded from above by −*ν*(*N*/*x*)^−*γ*^*s*_2_(*x*) and the second by −*νs*_1_(*x*), for some positive constant *ν*>0. It follows that

$$\begin{array}{rl} \frac{1}{dt}E[M(t+dt)-M(t)|F_{t}] & \leq -\nu N^{-\gamma }\left(\sum_{x:Y_{t}(x)=2}x^{\gamma }s_{2}(x)+\sum_{x:Y_{t}(x)=1}N^{\gamma }s_{1}(x)\right)\\ & \leq -\nu N^{-\gamma }M(t)\leq -\frac{\nu }{2}N^{-\gamma }(M(t)+1), \end{array}$$

on the event *M*(*t*)≥1. We introduce the process $Z(t)=\log(M(t)+1)+(\nu /2)N^{-\gamma }t$, and get

$$\frac{1}{dt}E[Z(t+dt)-Z(t)|F_{t}]\leq 0.$$

Observing that the extinction time *T*_ext_ of the process *Y* is also the first passage below one for the process *M*, we get that (*Z*(*t*): 0≤*t*≤*T*_ext_) is a positive supermartingale convergent to (*ν*/2)*N*^−*γ*^*T*_ext_. Using the optional stopping theorem, we deduce that $E[Z(T_{\mathrm{ext}})]\leq Z(0)$, and therefore

$$E[T_{\mathrm{ext}}]=\frac{2N^{\gamma }}{\nu }E[Z(T_{\mathrm{ext}})]\leq \frac{2N^{\gamma }}{\nu }\log(M(0)+1).$$

Initially every vertex is ready, and hence $M(0)=\sum_{x=1}^{N}s_{1}(x)∼N/(1-2\gamma ).$ We infer that the expectation of *T*_ext_ grows at most like a constant multiple of $N^{\gamma }\log N$ and the second part of theorem 2.1 is proved.

## Footnotes

1 We note, however, that network dynamics depending on the states of the nodes, are studied under the name adaptive, or coevolutionary, networks in the non-rigorous literature; see for example [19–22], and [23] for a rigorous example.

## Data accessibility

No data was used or generated for this research.
